# Prospects and perspectives: inferring physiological and regulatory targets for CAM from molecular and modelling approaches

**DOI:** 10.1093/aob/mcad142

**Published:** 2023-09-24

**Authors:** Methawi Chomthong, Howard Griffiths

**Affiliations:** Department of Plant Sciences, University of Cambridge, Cambridge, CB2 3EA, UK; Department of Plant Sciences, University of Cambridge, Cambridge, CB2 3EA, UK

**Keywords:** Transcriptome analysis, gene regulatory networks, hydraulic conductance, succulence, metabolic control, circadian systems

## Abstract

**Background and Scope:**

This review summarizes recent advances in our understanding of Crassulacean Acid Metabolism (CAM) by integrating evolutionary, ecological, physiological, metabolic and molecular perspectives. A number of key control loops which moderate the expression of CAM phases, and their metabolic and molecular control, are explored. These include nocturnal stomatal opening, activation of phosphoenolpyruvate carboxylase by a specific protein kinase, interactions with circadian clock control, as well as daytime decarboxylation and activation of Rubisco. The vacuolar storage and release of malic acid and the interplay between the supply and demand for carbohydrate reserves are also key metabolic control points.

**Future Opportunities:**

We identify open questions and opportunities, with experimentation informed by top-down molecular modelling approaches allied with bottom-up mechanistic modelling systems. For example, mining transcriptomic datasets using high-speed systems approaches will help to identify targets for future genetic manipulation experiments to define the regulation of CAM (whether circadian or metabolic control). We emphasize that inferences arising from computational approaches or advanced nuclear sequencing techniques can identify potential genes and transcription factors as regulatory targets. However, these outputs then require systematic evaluation, using genetic manipulation in key model organisms over a developmental progression, combining gene silencing and metabolic flux analysis and modelling to define functionality across the CAM day–night cycle. From an evolutionary perspective, the origins and function of CAM succulents and responses to water deficits are set against the mesophyll and hydraulic limitations imposed by cell and tissue succulence in contrasting morphological lineages. We highlight the interplay between traits across shoots (3D vein density, mesophyll conductance and cell shrinkage) and roots (xylem embolism and segmentation). Thus, molecular, biophysical and biochemical processes help to curtail water losses and exploit rapid rehydration during restorative rain events. In the face of a changing climate, we hope such approaches will stimulate opportunities for future research.

## INTRODUCTION

**Fig. 1.**
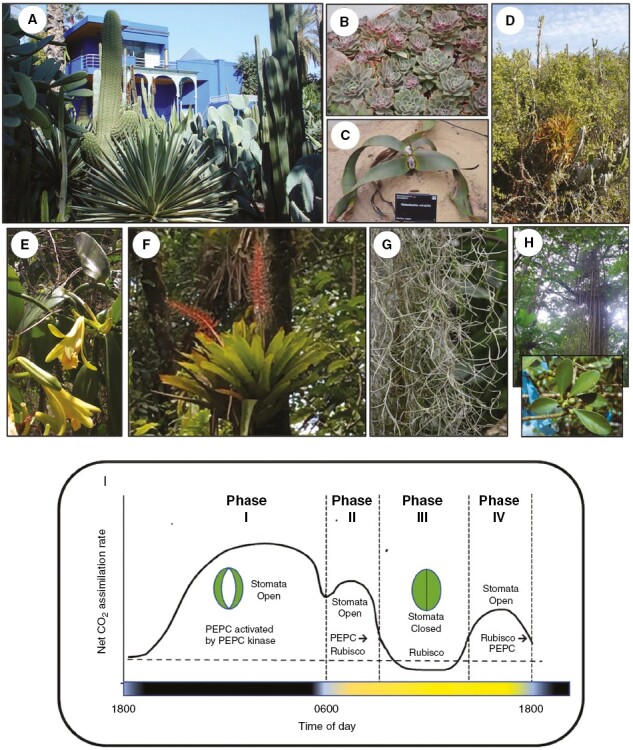
Diversity in succulent CAM lifeforms and a typical gas exchange profile. Terrestrial CAM plants: (A) massive stem succulent convergence in cacti (left) and euphorbia (right) species, with agave (centre) (Jardin Majorelle, Marrakesh); (B) *Echeveria elegans* (Crassulaceae); (C) *Welwitschia mirabilis* (Gnetophyta) (both Cambridge Botanic Garden); (D) Valley Bushveld or Thicket, Eastern Cape, dominated by *Portulacaria afra* and *Aloe* spp. Epiphytic CAM plants: (E) *Vanilla planifolia* (Orchidaceae), a hemi-epiphytic vine; (F) *Aechmea fendleri* (Bromeliaceae), a threatened montane epiphyte (both Trinidad); (G) *Tillandsia usneoides* (Bromeliaceae), Spanish moss, with marked vegetative reduction (Cambridge Botanic Garden); (H) *Clusia minor* (Clusiaceae), a hemi-epiphyte – canopy roots with seeds and leaves (inset), Trinidad. (I) Net CO_2_ assimilation highlighting defined phases of CAM (Osmond, 1978) and associated stomatal aperture status and carboxylation processes across the progression from night to day. All photographs taken by H.G.; diagram template provided by Rowan Sage.

The enduring interest in Crassulacean Acid Metabolism (CAM) extends well beyond the characteristic cellular or tissue succulence associated with the many striking leaf and stem succulent lineages, found across contrasting semi-arid habitats (Fig. 1). The co-ordinated evolution of traits has led to remarkable global diversity, with multiple independent origins of CAM, particularly in the last 5 million years, accounting for at least 6 % of extant angiosperm species (Dodd *et al*., 2002; Borland *et al*., 2018; Edwards, 2019; Wai *et al*., 2019; Gilman *et al*., 2023; Sage *et al*., 2023). The appeal for researchers lies partly in this numerical and ecological diversity of CAM lifeforms, and opportunities for the integration of phylogeny, ecophysiology, biochemistry, molecular biology and integrative modelling (Osmond *et al*., 1982; Chomthong and Griffiths, 2020; Winter and Smith, 2022). Additionally, the complexity and physiological plasticity of associated CAM phenotypic processes demands attention across day and night (Fig. 1; Dodd *et al*., 2002; Borland *et al*., 2009; Edwards, 2019).

A range of CAM lifeforms are illustrated in Fig. 1, with terrestrial representatives spanning massive leaf and stem succulents, including cacti, agave and euphorbia species (Fig. 1A) and more herbaceous succulents, such as many of the Crassulaceae (Fig. 1B). Over 60 independent origins of CAM (Gilman *et al*., 2023, Sage *et al*., 2023) range across lycopods, ferns, gymnosperms and angiosperms, with this diversity represented here by the gnetophyte *Welwitschia mirabilis* (Fig. 1C). When rainfall is relatively predictable on an annual basis, allowing tissue rehydration and storage, some semi-desert habitats can be dominated by CAM species, such as the Sonoran Desert Saguaro, the ‘Succulent Karoo’ in southern Africa or the *Portulacaria afra* (Didiereaceae) thicket in Eastern Cape, South Africa (shown in Fig. 1D). Many species in these habitats (including *Portulacaria*) are able to induce CAM in response to environmental stressors such as water deficits (Winter, 2019), and as notably identified by Klaus Winter in *Mesembryanthemum crystallinum* (Aizoaceae) following salinity stress (Winter and von Willert, 1972; Cushman, 2001; Winter and Holtum, 2007; Cushman *et al*., 2008).

The diversity and potential biomass of CAM plants found as epiphytes in tropical forests was highlighted by Winter (1985), including the largest family of CAM plants (Orchidaceae: Silvera *et al*., 2009; Gilman *et al*., 2023), here illustrated by *Vanilla planifolia* (Fig. 1E). Another major neotropical family is the Bromeliaceae, with over 3000 terrestrial and epiphytic species, with around 40 % demonstrating CAM activity (Crayn *et al*., 2015), with the proportion of epiphytes with CAM increasing along a gradient of decreasing rainfall (Griffiths and Smith, 1983). The occurrence of CAM in higher altitude, tropical montane forests (Pierce et al., 2002) is exemplifed by *Aechmea fendleri* (Fig. 1F), potentially under threat of climate change (Males *et al*., 2023). The marked vegetative reduction of the bromeliad leaf rosette seen in *Tillandsia usneoides* (Fig. 1G) has allowed this to become one of the most widespread of CAM plants, with a distribution ranging from Virginia, USA, to Argentina and tissues also providing a marker for water vapour inputs and exchange (Helliker, 2014). The final CAM life-form represented in this brief survey are the aerial roots of the woody, hemi-epiphytic strangling fig, *Clusia minor* (Fig. 1H, with inset showing fruits), with the comparative analysis of the Clusiaceae continuing to provide insights into the origins of CAM and leaf functional traits (Leverett *et al*., 2023*a*, *b*).

**Fig. 2.**
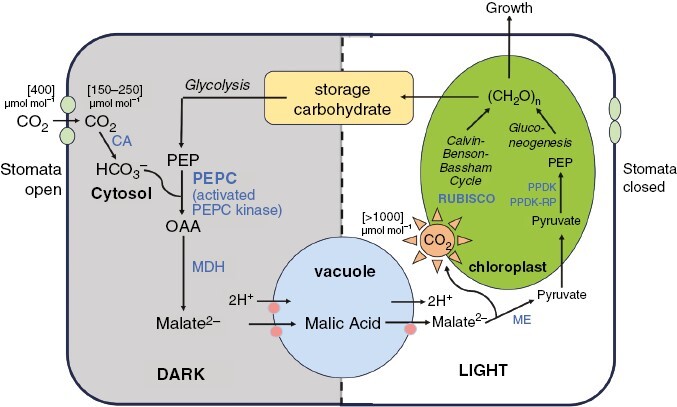
Schematic showing key features of Crassulacean Acid Metabolism (CAM). Primary carbon fluxes across dark and light conditions in mesophyll cells, with associated carboxylation and decarboxylation enzymes indicated in blue. At night, CO_2_ assimilation is mediated via cytosolic phosphoenolpyruvate carboxylase (PEPC, activated by phosphorylation via PEPC kinase) using carbon skeletons mobilized via glycolysis, leading to malate^2−^ synthesis, with malic acid accumulating in the vacuole via an aluminium-activated malate transporter, ALMT) and vacuolar ATPase/pyrophosphatase H^+^ transport system. In the subsequent light period, malic acid efflux (likely through the tonoplast dicarboxylate transporter, tDT: J. Andrew C. Smith, personal communication) is followed by decarboxylation (here illustrated by the cytosolic NADP-ME pathway). High concentrations of CO_2_ are generated internally and refixed by Rubisco in association with the Calvin–Benson–Bassham cycle, in chloroplasts. Carbon skeletons are recovered using gluconeogenesis, and carbohydrates are synthesized usually as chloroplastic starch/glucan (or vacuolar hexose in some species), and partitioned as reserves or exported for growth (Antony *et al.*, 2008). Diagram represents the chloroplastic decarboxylation pathway (after Winter and Smith, 2022), informed by enzyme localizations shown by Winter et al. (1982) and Lim *et al*. (2019). Some CAM systems utilize a cytosolic MDH and phosphoenolpyruvate carboxykinase decarboxylation pathway to generate PEP; mitochondrial enzymes may also be associated during primary carboxylation (particularly in terms of malate exchange) and decarboxylation (Dever *et al.*, 2015; Winter and Smith, 2022). CA, carbonic anhydrase; [CH_2_O], sugars/carbohydrate; MDH, malate dehydrogenase; ME, malic enzyme; OAA, oxaloacetate; PPDK, pyruvate, Pi dikinase; PPDK RP, PPDK regulatory protein.

The other key feature of CAM, also associated with these succulent traits, and visualized as a typical net CO_2_ exchange profile (Fig. 1I), is the reduced transpirational water loss associated with nocturnal stomatal opening, low stomatal densities and a steep diffusion gradient for CO_2_ uptake (Fig. 2). At night, phosphoenolpyruvate carboxylase (PEPC), with a high affinity for substrate HCO_3_^−^, is activated by phosphorylation associated with a specific PEPC kinase expressed *de novo* (Fig. 2; Hartwell *et al*., 1996, 1999; Boxall *et al*., 2017) leading via the reduction of oxaloacetate to malate^2−^ synthesis, which is defined as Phase I of CAM (Fig. 1I; Osmond, 1978). The mechanism of malic acid accumulation is shown in Fig. 2, with transport across the tonoplast into a large central vacuole coupled between transporter proteins and a combination of vacuolar ATPase and pyrophosphatase H^+^ pumps (Lim *et al*., 2019; Winter and Smith, 2022). This leads to the stoichiometric accumulation of 2H^+^:1mal^2−^:1CO_2_ (Fig. 2), allowing the magnitude of CO_2_ assimilation to be determined experimentally between dawn and dusk via the change in leaf-sap titratable acidity or enzymatic malate assays, as well as gas exchange profiles (Fig. 1I; Osmond, 1978).

At dawn, there is often a transient burst of CO_2_ assimilation known as Phase II, when PEPC is initially active (becoming sensitized to malate inhibition following dephosphorylation), and Rubisco is being activated in the light (Fig. 1I; Maxwell *et al*., 2002). During Phase III, net CO_2_ uptake is reduced as stomata close (Fig. 1I), prompted by both circadian control and the increase in internal CO_2_ generated during malate decarboxylation (Fig. 2; Chomthong and Griffiths, 2020). Elevated CO_2_ concentrations occur within mesophyll tissues during Phase III (>1000 mmol mol^−1^: Fig. 2), and often result in CO_2_ retrodiffusion and leakage (Fig. 1I). The processes in Fig. 2 illustrate chloroplast processes associated with decarboxylation by NADP-malic enzyme (NADP-ME) (with other variants also utilizing plastidic and mitochondrial elements: Lim *et al*., 2019; Winter and Smith, 2022). Under the elevated CO_2_ concentrations, Rubisco operates at a high operating efficiency, leading to suppressed oxygenase activity and photorespiration. Assimilation via the Calvin–Benson–Bassham cycle, together with regeneration of hexoses via plastidic pyruvate orthophosphate dikinase (PPDK) (Lim *et al*., 2019), allows carbohydrate reserves to be recharged and carbon export for growth (Fig. 2). During Phase IV, for well-watered plants, stomata may re-open and direct C_3_ carboxylation resumes (Osmond, 1978).

These physiological adaptations and the associated lower transpirational water loss at night translate into ecological advantages for the arid semi-desert habitats and tropical forest microclimates illustrated above (Fig. 1A–H; Kluge and Ting, 1978; Osmond *et al*., 1982; Winter, 1985). The mechanistic basis to these adaptations has stimulated investigations of the underlying molecular regulation (Cushman and Bohnert, 1999; Hartwell *et al*., 1999, 2016; Cushman *et al*., 2008). The plasticity in form and function associated with CAM phenotypes, and the associated physiology, ecology and molecular phylogeny continue to stimulate research approaches (Yang *et al*., 2015; Hartwell *et al*., 2016; Edwards, 2019, 2023; Heyduk *et al*., 2019*b*, 2023; Winter, 2019; Leverett *et al*., 2023*b*; Sage *et al.*, 2023).

The degree of succulence and hydration has been associated with the magnitude and length of the transient gas exchange phases (II, IV) which flank the nocturnal uptake (Phase I) and daytime decarboxylation (Phase III) (Fig. 1I; Griffiths *et al*., 2008; Owen and Griffiths, 2013; Owen *et al*., 2016; Males and Griffiths, 2018). The diversity in strong (obligate) CAM succulent morphologies ranges from the distinct rind of chlorenchyma and internal hydrenchyma found in the massive leaf and stem succulents (e.g. cacti, agave and euphorbia: Fig. 1A) to the homogeneous chlorenchyma found in the Crassulaceae (e.g. *Echeveria*: Fig. 1B, and *Kalanchoë* spp.) and the vegetative reduction seen in the strands of *Tillandsia usneoides* (Fig. 1G; Males and Griffiths, 2018; Chomthong and Griffiths, 2020). The phylogenetic distribution and independent origins of CAM in so many families (Edwards, 2019; Gilman *et al*., 2023) also leads to diversity in terms of the degree of CAM expression and induction in species ranging from annual and perennial herbs to the woody trees in the genus *Clusia* (Fig. 1H; Borland and Griffiths, 1990; Borland *et al*., 1992; Haag-kerwer *et al*., 1992; Dodd *et al*., 2002; Edwards, 2019; Leverett *et al*., 2023*b*) and *Aloidendron* (Grey *et al.*, 2022). The *de novo* induction of CAM triggered by abiotic stress in species such as *Mesembryanthumum crystallinum* (Adams *et al*., 1998; Taybi and Cushman, 1999; Cushman, 2001; Winter and Holtum, 2007) or in association with leaf development in *Kalanchoë* species (Hartwell *et al*., 2016) continues to provide molecular insights into the regulatory control of CAM.

From a popular perspective, the commercial dominance of pineapple (*Ananas comosus*) (Griffiths, 2023), as well the multiple economic uses of the Agavaceae, and huge interest horticulturally in the Orchidaceae, Cactaceae and Bromeliaceae, provide unique attractors. The focus of this review is to consider opportunities for future research on CAM, in the context of possible environmental threats for their semi-arid habitats posed by increasing climatic extremes.

The fundamental physiological mechanisms which control the inverted cycle of stomatal opening, and many of the nocturnal CO_2_ assimilation and daytime reassimilation processes, still elude a precise molecular definition. The diffusive constraints imposed by succulent cells and tissues, and hence low mesophyll conductances, are still debated regarding whether they are acting as cause or effect for the development of CAM (Leverett *et al*., 2023*b*; Heyduk *et al*., 2023). Whether all succulent plants demonstrate some degree of CAM activity is increasingly under discussion, as more detailed phylogenetic studies posit ever-earlier origins for the pathway (Heyduk *et al.*, 2019b, 2023; Gilman *et al*., 2023; Sage *et al*., 2023). Variations in leaf anatomy, whether enlarged chlorenchyma cells or chlorenchyma tissue with integrated or isolate d hydrenchyma, and origins of leaf venation, further complicate standardization (Ogburn and Edwards, 2013; Males and Griffiths, 2018). The capacity for storage of water within succulent tissues (e.g. leaf or stem) provides an ecological benefit for plants with limited, but regular water supplies, in both semi-arid desert environments and the epiphytic niches in tropical rainforests (Winter, 1985). Major questions remain regarding the gradual metering of water from these tissues to support the low nocturnal rates of transpiration, and requirement for rapid recharge and rehydration when precipitation events occur (Griffiths, 2013).

The progressive development of leaf thickness and associated changes in venation during the evolution of CAM had implications for the supply and demand of key resources, namely CO_2_ (mesophyll conductance in chlorenchyma) *and* H_2_O (hydraulic conductances in xylem and succulent tissues) (Griffiths, 2013; Ogburn and Edwards, 2013; Edwards, 2019). Building from this initial morphological tenet (Edwards, 2019), the central molecular and biochemical mechanisms which have been adapted or co-opted to allow the evolution and expression of CAM in so many lineages also now require a clearer definition. Whether there is a core temporal switch associated with a change in circadian expression, and a secondary response to physiological water stress, remain to be clarified at the molecular level for contrasting origins of CAM (Lim *et al*., 2019). However, the extent that expression of the CAM cycle can be modified by environmental conditions or metabolic feedback certainly suggests that changes in circadian expression are closely related to CAM induction and expression (Borland and Griffiths, 1997; Borland *et al*., 1999; Dever *et al*., 2015; Hartwell *et al*., 2016; Boxall *et al*., 2017, 2020).

The aim of this review is to consider some of the latest approaches incorporating physiological and molecular measurements, and their use in developing predictive models to explain CAM physiology, with a particular focus on hydraulic supply and demand. By developing a deeper quantitative understanding of the regulatory processes conferring CAM physiological plasticity, these more robust methods will allow the future opportunities for CAM research to be assessed, and allow predictions of ecological resilience in the face of a changing climate. We will consider various physiological modules which define key regulatory loops of the CAM cycle leading to the well-defined CAM phases (Osmond, 1978). These include stomatal opening at night, and activation of PEPC by a specific protein kinase at night, and interactions with circadian clock controls (Hartwell *et al*., 1996, 1999; Nimmo, 2000; Dodd *et al*., 2002), as well as decarboxylation and activation of Rubisco by day (Maxwell *et al*., 2002; Griffiths *et al*., 2007, 2008). The vacuolar storage and release of malic acid and the interplay between the supply and demand for carbohydrate reserves which fuel the CAM cycle are also key metabolic control points (Antony *et al.*, 2008; Borland *et al.*, 2016; Ceusters *et al.*, 2021). To initiate this review, we focus on recent data outputs which provide an opportunity to infer these core control mechanisms, and then summarize quantitative approaches which could help to identify likely physiological and genetic regulatory components conferring plasticity in expression of CAM phases across so many contrasting lineages.

### Modern genomics data and genetic manipulation tools underpin future directions for CAM researchers

In pursuit of a molecular definition of the regulatory processes leading to the evolution of CAM, the recent detailed analysis of CAM genomes provides a lens through which we may focus on orthologues and transcriptional control elements of key CAM genes (Ming *et al.*, 2015; Brilhaus *et al.*, 2016; Yang *et al.*, 2017; Wai *et al.*, 2019; Heyduk *et al.*, 2019a). Other studies taking a comparative ‘multi-omics’ approach provide additional insights into regulatory relationships in different CAM lineages (Abraham *et al*., 2016; Chiang *et al*., 2016; Zhang *et al*., 2016; Yin *et al*., 2018; Heyduk *et al*., 2019*b*). These studies have provided lists of candidate CAM-specific transcription factors (TFs) which could mediate CAM in obligate CAM species (*Kalanchoe fedtschenkoi* and *Agave americana*) (Moseley *et al*., 2018; Yin *et al*., 2018) and mediate the transition from C_3_ to CAM mode in facultative CAM species (*Mesembryanthemum crystallinum* and *Talinum triangulare*) (Brilhaus *et al.*, 2016; Amin *et al.*, 2019; Maleckova *et al.*, 2019). Third, additional layers of control are also being explored at the level of less conventional post-transcriptional regulation via microRNAs (miRNAs) as well as long non-coding RNAs (lncRNAs), which could function competitively with endogenous RNAs to alter the expression profile of key CAM pathway genes such as those for PEPC and PPDK (Yang *et al*., 2015; Wai *et al*., 2017; Bai *et al*., 2019). Research into pineapple identified several CAM genes being potentially regulated by miRNA or lncRNA (Wai *et al*., 2017; Bai *et al*., 2019). Transgenic manipulation experiments with RNA silencing approaches have been used to investigate the relationships between different genes in *Kalanchoë* species (Dever *et al*., 2015; Boxall *et al*., 2017, 2020). Finally, new molecular models are emerging, such as in the Portulacineae (Ferrari *et al*., 2021; Gilman *et al*., 2022; Edwards, 2023).

The comparative analysis of transcriptomes has identified genes which specifically alter expression patterns, whether seen as an inverted expression profile, shift in phase between C_3_ and CAM, or are responding to the CAM cycle (Yang *et al*., 2017; Moseley *et al*., 2018). These genes are thought to be those most likely to be synchronizing mesophyll processes. In addition to the comparison between C_3_ and CAM, the comparative genomics approach can be adopted between different species within the same family, with examples from orchids and pineapples (Ming *et al*., 2015; Zhang *et al*., 2016). An analysis of orchid genomes suggested that transcriptional control of carboxylation [*PPC* and *PPCK* genes for, respectively, PEPC and phosphoenolpyruvate carboxykinase (PEPCK)] and decarboxylation (PPDK) was coupled to the evolution of CAM in 13 orchid species (Zhang *et al*., 2016). Second, transcriptomic analysis of two cultivated pineapple varieties and one wild pineapple relative showed that CAM-related genes demonstrated an inverse diel expression pattern in photosynthetic tissues as compared to non-chorophyllous tissue (Ming *et al*., 2015). With the available pineapple genome data, they also suggested that CAM evolved not by gene duplication but through the modified expression of existing C_3_ genes. They also associated the circadian clock as a potential mechanism controlling CAM-specific gene expression, identifying putative *cis*-regulatory elements in key genes associated with those CAM genes that showed differential expression (Ming *et al*., 2015).

Insights for the origins of facultative CAM and expression of the C_4_ pathway have arisen from analysis of the *Portulaca amilis* genome (Gilman *et al*., 2022; Edwards, 2023). Here, CAM has been found to be ancestral within the Portulacineae, and gene expression network analysis identified a co-expression module associated with genes for PEPC and starch metabolism, together with circadian regulatory motifs (consistent with Yang *et al*., 2017; Wai *et al*., 2019). Also, the expression of C_4_ activity has been derived from diurnal CAM elements (Gilman *et al*., 2022).

### Recent advances in the molecular regulation of cellular metabolism

Carbohydrate and malate metabolism also show contrasting patterns between CAM and C_3_, since the inverse cycle of CAM requires carbohydrate reserves to accumulate to fuel the nocturnal CO_2_ fixation process (Osmond, 1978), with a different pathway for starch processing (Borland *et al*., 2016; Boxall *et al*., 2020). A comparison of C_3_ (*Arabidopsis*) and CAM (*K. fedtschenkoi*) showed that carbohydrate processing was differentially regulated in terms of the timing of expression (Yang *et al*., 2017). In addition to the changes in the timing of gene expression of genes associated with carbohydrate processing, network analysis also identified a specific role for trehalose-6-phosphate synthase and an invertase, which could control the partitioning of carbohydrates both to fuel the CAM pathway and to export reserves for growth (Borland *et al*., 2016; Yang *et al*., 2017). In pineapple (which primarily uses soluble sugars and starch as carbohydrate currencies), putative orthologues of chloroplast and vacuolar hexose transporters show a pronounced diel pattern of expression (Antony *et al.*, 2008; Borland *et al.*, 2016). In terms of malate accumulation, a specific channel (*ALMT*) seems to have been upregulated during the evolution of CAM in both pineapple and *Talinum* (Brilhaus *et al.*, 2016; Wai and VanBuren, 2018). Finally, for *Mesembryanthemum crystallinum* during the induction of CAM, Cushman *et al*. (2008) identified increased transcripts of a chloroplast carbohydrate transport gene associated with higher glucose-6-phosphate:phosphate translocator (*GPT2*) activity.

A correlation between circadian and metabolic control as also been demonstrated for both carboxylation (PEPC) and decarboxylation (mitochondrial NAD-malic enzyme, *NAD-ME*), with transgenic plants displaying altered timing of circadian clock genes and disrupted metabolic cycling (Dever *et al*., 2015; Boxall *et al*., 2017, 2020). For the carboxylation pathway, the regulation of PEPC has been recently investigated using two recent RNA silencing approaches. When *PEPCK1* was silenced in *K. fedtschenkoi*, the phosphorylation of *PPC* at night was reduced, together with a 66 % reduction in CO_2_ uptake at night, and the circadian rhythm of CO_2_ fixation associated with CAM collapsed to arrhythmia in the PPCK1 RNAi line (Boxall *et al*., 2017). When *PPC* was silenced in *K. laxiflora* using RNA interference (RNAi) (Boxall *et al*., 2020) night-time CO_2_ uptake ceased, and stomata reverted to daytime opening with all CO_2_ fixation in the light. There were pleiotropic effects on *PPCK1* transcripts, which were reduced at night but increased at the beginning of the light period, whereas expression levels of the other two detectable PPCK genes (*PPCK2* and *PPCK3*) were increased at night and daytime, respectively (although they are not thought to participate in the CAM cycle). In addition, although the transcript abundance of core circadian clock genes increased (such as Timing of Chlorophyll *a*/*b* binding protein, *TOC1*), gas exchange became arrhythmic under continuous light, temperature and humidity = LL conditions (stands for Light Light in day-night cycle) (Boxall *et al*., 2020).

Reducing the expression of genes associated with decarboxylation (NAD-ME and PPDK) also led to the loss of dark CO_2_ fixation associated with CAM, with plants compensating with daytime assimilation (Dever *et al*., 2015). Again, there were knock-on effects on the expression of genes associated with the CAM cycle, including *PPC*, and those associated with carbohydrate metabolism including *PPDK* (in the NAD-ME mutant), as well as glucan water dikinase and *GPT*.

Initial transgenic manipulation approaches did not target carbohydrate use in CAM directly; the overall carbohydrate balance was affected when PPCK and the decarboxylation pathway were reduced (Dever *et al*., 2015; Boxall *et al*., 2017). Starch accumulation by day was reduced, thereby reducing carbon skeletons available at night for CO_2_ assimilation (Osmond, 1978), but the highest levels of sucrose were associated with transcripts for a circadian response-regulator (Boxall *et al*., 2017). This provided more evidence for the potential connection between carbohydrate supply and circadian clock expression (Borland and Griffiths, 1997; Borland *et al*., 1999; Boxall *et al*., 2017; Gilman *et al*., 2022). Subsequently more specific studies have developed RNAi lines, either for plastidic starch phosphorylase (*PHS1*: Ceusters *et al*., 2021) or phosphoglucomutase (*PGM*: Hurtado-Castano *et al*., 2023). These have consolidated insights into the biochemical and molecular regulation of CAM evolution, with a reversal to those in controlling starch partitioning in the C_3_ pathway (Boxall *et al*., 2020). In transgenic CAM plants, transcripts for genes controlling the amylolytic pathway (C_3_) were upregulated as compared to the phosphorolytic pathway normally associated with starch storage during CAM (Borland *et al*., 2009, 2016; Ceusters *et al*., 2021). Also, starch degradation was not required to drive stomatal opening in the dark in the *PGM* mutant (Hurtado-Castano *et al*., 2023).

### Understanding temporal controls on the CAM pathway

The most complete explanation for temporal control is associated with PEPC, which is phosphorylated by a nocturnally expressed enzyme, PEPCK (Hartwell *et al*., 1999). The phosphorylated form of PEPC has lower sensitivity to malate inhibition, thus sustaining carboxylation at night. The regulation of the decarboxylation pathway is, however, less clear in CAM species. In C_3_ and C_4_ species, the enzyme PPDK is active in the dephosphorylated form whilst its phosphorylation and dephosphorylation reactions are catalysed by the same bi-functional enzyme, PPDK-regulatory protein (PPDK-RP) (Chastain *et al*., 2002; Om *et al*., 2022). This regulatory relationship between PPDK-RP and PPDK has not been verified in CAM species. The most direct evidence is from timeseries immunoblotting in *K. fedtschenkoi* which detected the dephosphorylated form of PPDK in the light (Dever *et al*., 2015), whereas in a CAM-deficient mutant of *K. laxiflora* (rPPC1-B line), PPDK remained phosphorylated and inactive and was not dephosphorylated in the light period (Boxall *et al*., 2020). This suggested that the phosphorylation/dephosphorylation reaction may be partly responsible for the diurnal activation of PPDK in *K. fedtschenkoi*. Nonetheless, the conditions that facilitate the alternating function of PPDK-RP between kinase and phosphorylase are not known for CAM. In addition, there is another level of complexity due to the neofunctionalization of PPDK, whereby two-thirds of this protein is neofunctional in the cytosol, whereas the remaining third is in chloroplasts for *K. fedtschenkoi* (Kondo *et al*., 2000). This ratio also varies in other members of Crassulaceae (Kondo *et al*., 2000). It is evident that activities of different compartments of CAM must be highly orchestrated (Borland *et al*., 2016; Chomthong and Griffiths, 2020; Ceusters *et al*., 2021).

We lack a detailed understanding of how the physiological expression of CAM is regulated directly in response to environmental and metabolic effectors, or may respond to prompts from circadian networks or other genetic regulators. For instance, the timing of changes in stomatal conductance and activation of carboxylation activities influence, respectively, the CO_2_ diffusive supply and demand. It is generally thought that the stomatal intercellular CO_2_ (Ci)-sensing pathway responds to both the reduction in Ci at night and the increase during decarboxylation by day (Borland and Griffiths, 1997; Drennan and Nobel, 2000; Ceusters *et al*., 2008; von Caemmerer and Griffiths, 2009) although some additional circadian control at night was evident in *PPC* knockdown plants (Boxall *et al*., 2020). The control of carbohydrate partitioning, with carbohydrate substrate supply associated with the magnitude of night-time CO_2_ assimilation and malic acid accumulation, correlates with the previous day’s light intensity (Nobel, 1988; Borland *et al*., 2016, 2018). Genetic manipulation experiments have demonstrated the complexity of these interactions, and how primary signals trigger upstream responses either due to metabolites or the circadian clock (Dever *et al*., 2015; Boxall *et al*., 2017, 2020). One specific example relates to the knockdown of the gene coding for the decarboxylation enzyme NAD-ME. In the NAD-ME knockdown line, the phosphorylated form of PPDK was detected throughout a 24-h period, implying the constitutive down-regulation of PPDK activity, metabolically downstream of NAD-ME activity (Dever *et al*., 2015).

The regulation of stomatal behaviour also brings an additional level of complexity – whether due to contrasting responses to light intensity/quality following CAM induction, or developmental stages (Lee & Assmann, 1992; Tallman *et al*., 1997; Gotoh *et al*., 2019; Hurtado-Castano *et al*., 2023). On the one hand, the stomata of facultative CAM species (*Mesembryanthemum crystallinum* and *Portulacaria afra*) were reported to not respond to light once induced into the CAM mode (Lee and Assmann, 1992; Tallman *et al*., 1997). On the other hand, obligate CAM species (*K. daigremontiana* and *K. pinnata*) still showed blue light-induced stomatal opening as commonly documented in C_3_ species (Gotoh *et al*., 2019). These contradictory observations provide a focus on the interplay between light cues with the CAM pathway. The extent that an inverse cycle of guard cell PEPC activity and malate metabolism could contribute to stomatal solute balance and stomatal opening at night has not been resolved (Santelia and Lawson, 2016; Males and Griffiths, 2017). Recent studies suggest a role for diurnal anion channel transcription in regulating stomatal opening at night (Lefoulon *et al*., 2020), and the absence of any role for guard cell starch accumulation (Hurtado-Castano *et al*., 2023). Additionally, increased transcripts associated with abscisic acid (ABA) synthesis and signalling before dawn suggested a possible role in triggering daytime stomatal closure (Abraham *et al*., 2016), although the direct effect of ABA on the inverted timing of CAM stomatal behaviour remains elusive (Holtum and Winter, 1982; Chu *et al*., 1990; Bastide *et al*., 1993; Dai *et al*., 1994; Taybi *et al*., 1995; Taybi and Cushman, 1999).

### Integrating plant hydraulic pathways and cell succulence

In a recent review, Chomthong and Griffiths (2020) called for an additional focus on the co-evolution of tissue succulence and CAM hydraulic relationships to characterize the contrasting theories defining cause or effect between water deficits and the development of CAM (Edwards, 2019). There have been a number of recent studies which have addressed the interplay between cell succulence and responses to water deficits (Fradera-Soler *et al*., 2022*a*, *b*) and implications for the evolution of CAM in the genus *Clusia* (Leverett *et al*., 2023*a*, *b*). Specifically, the historical importance of cell wall plasticity for succulent cell water relations (Murphy and Smith, 1998) has now been given a metabolic basis, with mannans and cell wall folding (Ahl et al., 2019), and plasticizers helping to facilitate cell-wall collapse during drought (Fradera-Soler *et al*., 2022*a*, *b*). These authors also identified convergent evolution in terms of contrasting cell-wall metabolic components in monocots (probable remobilisation of mannans) and the Caryophyllales (apoplastic pectin-rich mucilage) to facilitate cell-wall folding, hence conferring plasticity (Fradera-Soler *et al*., 2022*a*, *b*).

Another focus has been on the interplay between leaf venation density and leaf succulence (Heyduk, 2021; Jolly *et al*., 2021) and evolution of CAM (Leverett *et al*., 2023*a*, *b*). The requirements for succulent cells to increase hydraulic capacitance, as well as providing an appropriate volume for the storage of malic acid during CAM, have long been considered to represent possible drivers for the evolution of CAM (Edwards, 2019). Detailed analyses of succulent cell differentiation (water storage vs. chlorenchyma) for C_3_ and CAM species in the woody genus *Clusia* have suggested that the evolution of CAM and hydraulic capacitance are independent traits (Leverett *et al*., 2023*a*, *b*) which was also supported using the mechanistic model of Hartzell *et al.* (2018, 2021). Increased 3D vein density in more succulent (thicker) leaves has been associated with higher drought tolerance in C_3_*Yuccas* (Jolly *et al*., 2021) and also to support CAM in *Clusia* species (Leverett *et al*., 2023*b*), perhaps by improving hydraulic supply and recharge (Griffiths, 2013).

These studies have helped to develop the insights provided by Ogburn and Edwards (2013) on the contrasting mechanisms leading to 3D venation, whereby the development of succulent tissues is associated with vein placement throughout tissues and shifting from a planar, 2D, organization. First, it is important to recognize the difference between all-cell (homogeneous chlorenchyma) and storage succulents, with chlorenchyma confined to a photosynthetic rind surrounding larger leaves or stems (Males, 2017; Males and Griffiths, 2018; Chomthong and Griffiths, 2020), or interspersed hydrenchyma, between chlorenchyma, generally seen in the genus *Clusia* (Leverett *et al*., 2023*a*) and the Bromeliaceae (Males, 2017; Males and Griffiths, 2018). Second, succulent tissues increase hydraulic capacitance thereby contributing to avoidance of water deficits, and changes in venation which support the development of succulence provide an evolutionary advantage (Ogburn and Edwards, 2013; Edwards, 2019). Finally, we hope that researchers will take up the challenge to test the hypothesis that co-ordination of overall root–shoot hydraulic conductance is more important for rapid recharge and rehydration of succulent tissues, rather than the subsequent more gradual nocturnal metering of transpirational water loss during CAM (Griffiths, 2013; Chomthong and Griffiths, 2020).

Thus, CAM is dependent on complementary mechanisms in succulent tissues which balance low stomatal and mesophyll conductances (constraining CO_2_ uptake), and hydraulic conductances (both within xylem and across mesophyll tissues) to optimize water uptake, storage and transpiration (Edwards, 2019; Chomthong and Griffiths, 2020). These features contribute to sustaining the high tissue water contents and leaf water potentials (Ψ) typically associated with CAM leaves or cladodes, with Ψ normally around −1 MPa. The extreme sensitivity of CAM plant roots to water deficits is demonstrated by the observations that radial root shrinkage occurs around soil Ψ as low as −0.3 MPa, which helps to isolate terrestrially rooted CAM plants (Nobel, 1988; North *et al*., 2008). Physiological models need to account for the biophysical regulation of water flow from roots to shoots, and specifically the control exerted by aquaporins (Martre *et al*., 2002; Caldeira *et al*., 2014; Lu and Fricke, 2023) and interplay with hydraulic isolation and segmentation, needed to protect succulent shoot tissues (Nobel, 1988; Martre *et al*., 2002; Males, 2017).

In terms of modelling these processes, some models do utilize resistances associated with the water potential gradient across the soil–plant–tissue continuum (Bartlett *et al*., 2014; Caldeira *et al*., 2014; Hartzell *et al*., 2018, 2021). However, these approaches do not incorporate the mechanistic plasticity associated with physiological processes during the onset of, and recovery from, drought. These include root shrinkage, cavitation and segmentation, and then the relative roles of aquaporins and plasmodesmata for re-establishing, and then maximizing, water flows across root and shoot tissues (Martre *et al*., 2002; North *et al*., 2008; Males, 2017). The sensitivity of the root–shoot junction has been identified by Males (2017) as another key developmental transition between the xylem of roots (primarily vessels) and shoots (tracheids and low-diameter vessels), possibly helping to maximize root hydraulic flows when water is available (Griffiths, 2013). We also require a much better molecular framework to define the genes associated with the cell enlargement, and vein differentiation during the development of succulent tissues (Heyduk, 2021; Heyduk *et al* 2023), and their integration with the processes described above during seedling establishment and growth, relative to water availability.

### Molecular evolution of the CAM pathway

In terms of the evolution from C_3_ ancestral species, it has been hypothesized that key CAM genes were already present in the C_3_ system, but the regulatory components were rewired to result in the specific temporal characteristics of the CAM pathway (Cushman and Bohnert, 1999; Cushman *et al*., 2008; Ming *et al*., 2015). Analysis of the pineapple (*An. comosus*) genome showed that CAM in that species has evolved from re-wiring of existing C_3_ components through regulatory neofunctionalization of pre-existing genes (Ming *et al*., 2015), rather than by coding neofunctionalization (Wai *et al.*, 2017; Abraham *et al*., 2016; Chiang *et al*., 2016; Zhang *et al*., 2016; Yin *et al*., 2018; Brilhaus *et al.*, 2016; Heyduk *et al*., 2019*a*, *b*). Whole-genome duplication and single-gene duplication are common features in plant genome evolution. After duplication events, there may be wholesale losses of duplicate genes, in a process known as fractionation (Panchy *et al*., 2016). Alternatively, the duplicated genes that are retained within the genome can either partition the original gene function (i.e. subfunctionalization) or develop novel functions (neofunctionalization). The two types of neofunctionalization are regulatory neofunctionalization and coding functionalization. Regulatory neofunctionalization is a result of expression divergence which allows the protein encoded from the duplicated gene to function in a different temporal or spatial environment as compared to the protein encoded from the original gene. In contrast, coding neofunctionalization results in a novel protein function due to the gain-of-function mutation in the coding region of the duplicated gene (Hughes *et al*., 2014).

Key CAM genes are present in C_3_ species, but do not function in the primary CO_2_ fixation pathway as in CAM. For instance, the roles of β-carbonic anhydrase (β-CA) in C_3_ range across photosynthetic and non-photosynthetic tissues (Aubry *et al*., 2011) but despite changes in gene expression (Ming *et al*., 2015), there is no functional evidence that β-CA is crucial for catalysing the formation of HCO_3_^−^ for primary CO_2_ fixation in C_4_ or CAM (Lim *et al*., 2019). In C_3_ species, the enzyme PEPC has a range of functions, including provision of carbon skeletons for the Krebs cycle and the ammonium assimilation pathway (Miyao and Fukayama, 2003; Masumoto *et al*., 2010), maintaining malate homeostasis, and regulating stomatal conductance (Aubry *et al*., 2011). Thus additional analyses of transcriptional networks will help to identify specific gene neofunctionalization associated with CAM (Wai *et al*., 2015; Abraham *et al*., 2016; Chiang *et al*., 2016; Zhang *et al*., 2016; Yin *et al*., 2018; Brilhaus *et al.*, 2016; Heyduk *et al*., 2019*a*, *b*; Gilman *et al*., 2022).

To identify such rewiring of the gene network, duplication events followed by changes in the protein sequence and regulatory motifs provide evidence for the formation of novel regulatory relationships, which then dictate the distinct diel expression profiles of the CAM system. These genome rearrangements have been identified in multiple CAM species (Yang *et al*., 2017; Heyduk *et al*., 2019*a*). The presence of multiple paralogues of key CAM genes may have facilitated the neofunctionalization of the newly duplicated genes. For instance, there are five paralogous PEPC genes in the *K. fedtschenkoi* genome with a single copy being highly expressed specifically at night (Yang *et al*., 2017), as compared to four found in *Arabidopsis thaliana* and *Vitis vinifera* (unpublished phytozome search, https://phytozome-next.jgi.doe.gov/). However, evidence regarding functional changes in the regulatory motif in the promoter region of this duplicated PEPC gene is still lacking. In a different CAM model species, pineapple, there has been a search for regulatory motifs in promoter regions of key CAM genes which include *β-CA, PPC, PPCK*, *MDH, PEPCK* and *PPDK* (Ming *et al*., 2015). This study in pineapple searched for five known circadian clock-related motif sequences: a morning element, an evening element, CCA1-binding site, a G-box element and a TCP15-binding motif. They identified the presence of at least one of these five motifs for all of these genes of interest, with the exception of PPDK. However, care must be taken to infer functional associations purely from transcript abundance (Dever *et al*., 2015), and direct evidence for specific TFs and the conditions under which TF binding occurs has yet to be characterized functionally.

To address key questions for CAM, such as how enzymes and their related pathways are regulated to become active/inactive at the right time of the day–night cycle, we suggest that a combination of systems biology approaches are required. First, we require additional developments in quantitative models which integrate the various CAM control loops, and need to develop reliable predictive outputs, which are testable against data from physiological and molecular manipulations. Second, we need to use bioinformatic tools for handling large-scale transcriptomic datasets, and the development of various computational techniques to explore gene regulatory networks (GRNs) and TF activation which controls the expression of CAM Phases.

### Large-scale approaches

A recent review described recent advances in CAM modelling and the possibilities for any newly available ‘-omics’ data to augment our understanding of CAM (Chomthong and Griffiths, 2020). Some of the mechanistic models are based on flux balance analysis (Orth *et al*., 2010; Shameer *et al*., 2018; Töpfer *et al*., 2020), oscillatory models (Blasius *et al*., 1998; Bartlett *et al*., 2014; Hartzell *et al*., 2018, 2021) or a system dynamics model (Owen and Griffiths, 2013, Chomthong, 2023) (for details see section below). Data outputs from the manipulation of gene expression provide key inputs to validate the accuracy of predictions arising from such models, in terms of the goodness of fit to associated changes in CAM phase expression and metabolic activity (Dever *et al*., 2015; Boxall *et al*., 2017, 2020; Ceusters *et al*., 2021), particularly if validated by metabolic flux balance analysis (Orth *et al*., 2010; Cheung *et al*., 2014) augmented with ^13^C labelling (Ishihara *et al*., 2015). The advantage of a bottom-up approach is that it does not require prior knowledge about an entire regulatory network. In contrast, top-down approaches such as the construction of GRNs, protein–protein interaction networks and metabolic networks require genome (proteome/metabolome)-wide input data (Emmert-Streib *et al*., 2014). The challenge of the top-down approach is to connect the complete information back to the classical mechanistic understanding of the CAM system and distinguish between causation and correlation.

The ultimate aim would be to unite top-down and bottom-up approaches to define a more structured identification of potential gene knockdown/knockout targets for transformation, and the development of a wider range of transformation systems, such as *K. fedtschenkoi* and *K. laxiflora* (Hartwell *et al*., 2016; Liu *et al*., 2019). The physiological responses of such manipulated lines would allow the metabolic circadian, developmental and stress response control points to be identified, as well as improving the goodness of fit for re-parameterized models. In addition, models developed specifically for diverse modes of CAM expression could be used to explore the genetic and metabolic basis for the transition from C_3_ to CAM, and help to improve phylogenetic and evolutionary insights into the origins of CAM (Edwards, 2019) or inform CAM biodesign for the future (Lim *et al*., 2019).

### Large-scale physiological modelling

The higher water use efficiency associated with CAM species has been the basis for modelling the use of plants such as *Agave* and *Opuntia* in marginal lands as an alternative form of biofuel (Borland *et al*., 2009; Owen *et al*., 2016). To this end, field-scale modelling has been developed to predict CAM productivity in response to environmental fluctuations (Hartzell *et al*., 2021).

In parallel to the global-scale productivity modelling, progress on CAM metabolic modelling has been made continuously. Several modelling approaches address temporal orchestration and metabolite partitioning in CAM, which can be grouped into three categories. First, flux balance analysis has captured the complete metabolic network under stoichiometric constraints and optimization of the objective function (Orth *et al*., 2010; Shameer *et al*., 2018; Töpfer *et al*., 2020). Second, a category includes mechanistic models which incorporate simplified mathematical representations of the CAM circadian rhythm (Blasius *et al*., 1998; Bartlett *et al*., 2014; Hartzell *et al*., 2018). Third, a systems dynamic (SD) model representation of biochemical and physiological components of the CAM pathway has allowed the fine-tuning of key parameters (Owen and Griffiths, 2013). This latter approach has allowed the relative contribution to limitations such as stomatal sensitivity, carboxylation processes and vacuolar storage to predict the impact of succulence on gas exchange profiles (Owen and Griffiths, 2013).

This SD model can be mathematically expressed in its fundamental form of ordinary differential equations (ODEs) to develop more rigorous mathematical analyses. These equations take derivatives with respect to one independent variable. The term ‘ordinary’ distinguishes the approach from partial differential equations (PDEs), where the derivatives are taken with respect to multiple variables. Combining the SD model (Owen and Griffiths, 2013) with ODE approaches generates a model which can be parameterized and tested against experimental data (Chomthong, 2023). Thus, the emergence of genetic and physiological manipulation experiments can be integrated into these modelling tools to investigate the effect of perturbation on the system responses by comparing measured and predicted changes in expression of the canonical CAM pathway (Chomthong, 2023).

### Large-scale molecular modelling

The genome sequencing and transcriptome sequencing of the *K. fedtschenkoi* 256-Mb genome (diploid 2*n* = 34 chromosomes) provided a detailed analysis of CAM in the Crassulaceae (Yang *et al*., 2017). After genome assembly, the 30 964 protein-coding genes were predicted and annotated and they identified two distinct whole genome duplication events in *K. fedtschenkoi* based on analysis of the syntenic patterns. However, this is different from the scenario in the pineapple genome wherein the key CAM genes did not undergo duplication. Instead, the pineapple CAM genes are the same ancestral copies that are present in non-CAM grass species, but are more highly expressed in CAM tissue compared to non-photosynthetic tissues (Yang *et al*., 2017).

The transcriptome obtained by Yang *et al*. (2017) was used for gene co-expression analysis, cluster analysis and inferring the convergent evolution of key genes. A total of 25 gene co-expression modules were obtained from the weighted correlated network. Notably, the genes encoding β-CA, *PPC2*, *PPCK*, *MDH* and *ALMT6* were clustered into the night-time module (although PPC2 does not functionally activate PEPC expression: Boxall *et al*., 2017), whereas *PPDK-RP* was clustered into the daytime module (Yang *et al*., 2017). In addition to the gene co-expression module analysis, Yang *et al*. (2017) also performed cluster analysis. Clustering analysis returned 11 clusters with a zinc-finger protein *CONSTANS-like* gene as a central hub for a cluster containing *PEPC1* and *PPCK2* (although *PPCK1* is involved in *PPC* phosphorylation in this species; Boxall *et al*., 2017). The evening-element binding *REVEILLE* TFs acted as hubs in a cluster of *NADP-ME* genes (Yang *et al*., 2017), although NAD-ME is the major decarboxylase in this species (Dever *et al*., 2015).

The study by Yang *et al*. (2017) also attempted to identify the convergent evolution of CAM genes through the convergent diel expression profiles and the convergent amino acid changes in the protein-coding sequences of CAM-associated genes. Initially, the convergent diel expression profiles were created by comparing the timeseries gene expression profiles of *K. fedtschenkoi* (eudicot CAM), *An. comosus* (pineapple, monocot CAM) and *Ar. thaliana* (eudicot C_3_). The authors used three conditions to define the convergent diel expression profiles between these two CAM species. Based upon a timecourse analysis, this approach identified 54 genes with convergent diel expression profiles between *K. fedtschenkoi* and pineapple as opposed to the profiles of the C_3_ plant. These 54 genes include important genes with well-known functions such as phosphoenolpyruvate carboxylase kinase 1 (PPCK1), phototropin 2 (*PHOT2*) and heat shock protein 70 (*HSP70*). In a parallel analysis, protein sequence convergence was studied for *K. fedtschenko*i (Yang *et al*., 2017) but some of the genes they associated with carboxylation and decarboxylation may be redundant (Dever *et al*., 2015; Boxall *et al*., 2017). However, the extremely wide taxonomic divergence associated with this comparative analysis suggests that more targeted manipulation of the *K. fedtschenko*i genome (through specific gene silencing and associated transcriptome sampling) would provide a more focused approach today.

For a mechanistic understanding of gene regulation to be drawn from these newly available large-scale transcriptomic data requires GRN analysis. Such GRN approaches analyse highly complex interactions to infer transcriptional regulators and their respective target genes. The availability of the microarray and/or transcriptome data has opened up the way for the construction of GRNs by *inference* methods in various biological systems. The term *inference* indicates the nature of the resultant GRNs which are based on the interactions of genes calculated through the model structures rather than the direct evidence of transcriptional regulator binding to the promoter regions of the genes of interest. The GRN inference approach has been widely used in animal systems and subsequently has been adopted by the plant community (Emmert-Streib *et al*., 2014). Co-expression network analysis suggested the number of potential transcriptional regulators of CAM could in theory be as high as 1509 candidate genes from *Agave americana* (Yin *et al*., 2018), although the actual number of potential regulators in the CAM system is likely to be lower. Hence, the GRN inference approach has the potential to become a useful tool for addressing CAM questions, for example through using an algorithm such as the dynamic GENIE3 (dynGENIE3), which has been developed specifically to handle timeseries datasets (Huynh-Thu and Geurts, 2018). With the genome size of *K. fedtschenkoi*, the total number of possible combinations of regulatory relationships within the genome would be 958 738 332 interactions, which are impossible to calculate manually (Chomthong, 2023). With the dynamic GENIE3 algorithm, every combination of the regulatory relationships between potential transcriptional regulators and their corresponding target genes are ranked by statistical scores. In principle, the analysis could identify TF expression associated with the circadian clock, their CAM-associated gene and specific promoter sequence elements associated with that specific TF. Their paper identifies a number of TF targets within the promoter regions of key CAM genes. However, subsequent functional annotation and experimental manipulation is needed to identify the actual relationship between these predicted regulators and expression in CAM systems.

### Advanced experimental approaches for identifying the transcriptional regulation of CAM

Despite predictive power of the GRN approach, it relies heavily on transcriptome data. To complement the GRN predictions from computational methods, experimental data that capture the chromatin accessibility state will be required. This type of data can be obtained from complementary approaches such as DNase I hypersensitive sites sequencing (DNaseI-seq), micrococcal nuclease digestion with deep sequencing (MNase-seq), formaldehyde-assisted isolation of regulatory elements (FAIRE-seq) and chromatin immunoprecipitation (ChIP-seq). Alternatively, the assay for transposase-accessible chromatin with high-throughput sequencing (ATAC-seq) is the state of the art method with the fastest protocol yet requires the smallest number of pure nuclei compared to the other protocols previously mentioned (Tsompana and Buck, 2014). The ATAC-seq method relies on the highly active transposase enzyme Tn5 to insert the sequencing primers preferentially into the physically accessible regions of chromatin strands. By sequencing the transposase-accessible regions with next-generation sequencing, the sequencing reads can be aligned back to the genome to indicate the open regions across the genome.

Such sequencing could identify open chromatin regions and potentially recover TF footprints, given sufficient sequencing depth. The information would complement predictions from GRN inference and verify the roles of candidate transcriptional regulators and the conditions under which the promoter regions of target genes are accessible. This would be important for advancing our understanding of the gene regulatory landscape of CAM in the near future.

The first step in the ATAC-seq protocol is to purify nuclei from the tissue samples. For CAM species, there is currently no protocol and one option would be to modify the transgenic protocol developed for the RNAi method (Dever *et al*., 2015) or the sucrose-gradient sedimentation method, in combination with a spectral flow cytometer in order to purify CAM nuclei for subsequent sequencing (Lu *et al*., 2017; Bajic *et al*., 2018). It is to be expected that the combination of GRNs and chromatin accessibility will advance understanding of the gene regulatory landscape of CAM in the near future.

### Wither or whither for CAM: getting to the root of the problem

From a research perspective, it is clear that the field continues to advance dynamically, whether from a phylogenetic, ecological, physiological or molecular perspective, and that a wealth of younger researchers are continuing to develop novel insights and correlations. The challenge will be to integrate datasets operating at contrasting scales. The increasing availability of transcriptome data requires novel analytical systems to provide specific candidate regulatory targets, including TFs and their binding sites as effectors on multiple genes activating CAM processes, as well as developing methods for nuclei isolation and sequencing (Chomthong, 2023). Key questions remain to be answered, and it is likely that the convergent evolution of CAM was not a unified journey for contrasting lineages (Edwards, 2019, 2023; Chomthong and Griffiths, 2020; Gilman *et al*., 2022, 2023; Sage *et al*., 2023). These conclusions are justified by contrasting succulent tissues, with the diverse degrees of integration of chlorenchyma and hydrenchma found across succulent herbs to massive leaf and stem succulents (Males and Griffiths, 2017). For trees in the genus *Clusia*, it seems that the requirement for succulent tissues to support the CAM cycle is to some extent independent of the hydraulic capacitance, although there are convergent requirements to increase vein density to service hydraulic supply and demand (Leverett *et al*., 2023*b*).

Ecologically, CAM plants are to some extent under threat of climatic extremes: the reliance on predictable seasonal rainfall has been recognized as a key factor regulating CAM diversity and survival (Borland *et al*., 2009; Griffiths, 2013). The impact of increased climatic extremes (heat and drought) on CAM performance has recently been reviewed (Heyduk, 2022). Key questions have been raised regarding environmental interactions in a high CO_2_ world, including the extent that susceptibility or range expansion will differ across contrasting lineages, particularly for habitats ranging from semi-arid deserts to tropical forest epiphytes (Heyduk, 2022; Males *et al*., 2023). A specific example includes a recent popular report that *Opuntia* and other succulents are invading south-facing slopes in the Swiss and French alps (https://www.theguardian.com/environment/2023/feb/10/cacti-replacing-snow-on-swiss-mountainsides-due-to-global-heating) because of reduced snow cover associated with global warming. Other examples include an invasive *Kalanchoë* species (Herrando-Morrraira *et al*., 2020) and responsiveness of CAM to drought and elevated CO_2_ of CAM, relative to C_4_ (Yu *et al*., 2019), and the probable loss of CAM bromeliad epiphytes from montane tropical forests (Males *et al*., 2023). In general, it seems that opportunities for biofuel production associated with CAM are likely to increase in what were thought to be temperate climes (Owen *et al*., 2016; Hartzell *et al*., 2021). Elsewhere there are the threats to CAM biodiversity through collection from the wild and resale of orchids, bromeliads and cacti (Goettsch *et al*., 2015).

Physiologically, understanding the balance between circadian control (in regulating metabolism) and metabolic feedback (on clock timing of gene expression) regulation will continue to provide intriguing challenges for the CAM community, as highlighted above. We have also highlighted the ongoing discussions regarding the co-evolution of succulent tissues and CAM (Edwards, 2019; Chomthong and Griffiths, 2020; Leverett *et al*., 2023*b*), but feel that key questions (the root of the issue, as it were) relate to the physiological and molecular processes associated with the hydraulic conductance of root systems, which confer drought avoidance found in succulent leaves and stems above ground (North *et al*., 2008; Griffiths, 2013). Finally, we note that the availability of molecular tools, through gene knockdown or silencing, are helping to dissect these regulatory processes (Dever *et al*., 2015; Boxall *et al*., 2017, 2020; Liu *et al*., 2019). Thus, large-scale molecular datasets that can be analysed using computational tools can be coupled with advanced mechanistic and process-based models to compare predicted outputs with actual changes in the physiological expression of CAM associated with molecular targets. These integrated approaches will provide many challenges for CAM researchers in the future, but their application will help to resolve many of the evolutionary and ecological questions regarding the origins and future potential for such fascinating plant communities.

## FUNDING

MC was initially funded by a Scholarship from the The Institute for the Promotion of Teaching Science and Technology (IPST), a Thai State Agency. We are also grateful for financial support from the GCRF Collective Call: BBSRC BB/P027970/1 (TIGR2ESS) programme.
